# The value of completion axillary treatment in sentinel node positive breast cancer patients undergoing a mastectomy: a Dutch randomized controlled multicentre trial (BOOG 2013-07)

**DOI:** 10.1186/s12885-015-1613-2

**Published:** 2015-09-03

**Authors:** L. M. van Roozendaal, J. HW de Wilt, T. van Dalen, J. A. van der Hage, L. JA Strobbe, L. J. Boersma, S. C. Linn, M. BI Lobbes, P. MP Poortmans, V. CG Tjan-Heijnen, K. KBT Van de Vijver, J. de Vries, A. H. Westenberg, A. GH Kessels, M. L. Smidt

**Affiliations:** 1 Division of Surgical Oncology, Maastricht University Medical Centre, Maastricht, The Netherlands; 2 Division of Surgical Oncology, Radboud university medical centre, Nijmegen, The Netherlands; 3 Division of Surgical Oncology, Diakonessenhuis Hospital, Utrecht, The Netherlands; 4 Division of Surgical Oncology, Netherlands Cancer Institute - Antoni van Leeuwenhoek Hospital, Amsterdam, The Netherlands; 5 Division of Surgical Oncology, Canisius-Wilhelmina Hospital, Nijmegen, The Netherlands; 6 Department of Radiation Oncology, Maastricht University Medical Centre (MAASTRO clinic), Maastricht, The Netherlands; 7 Division of Medical Oncology, Netherlands Cancer Institute, Antoni van Leeuwenhoek Hospital, Amsterdam, The Netherlands; 8 Department of Radiology and Nuclear Medicine, Maastricht University Medical Centre, Maastricht, The Netherlands; 9 Department of Radiation Oncology, Radboud university medical centre, Nijmegen, The Netherlands; 10 Division of Medical Oncology, Maastricht University Medical Centre, Maastricht, The Netherlands; 11 Department of Pathology, Netherlands Cancer Institute, Antoni van Leeuwenhoek Hospital, Amsterdam, The Netherlands; 12 Department of Medical and Clinical Psychology, Tilburg University, Tilburg, The Netherlands; 13 Radiation Oncology, Arnhem Institute for Radiation Oncology, Arnhem, The Netherlands; 14 Department of Clinical Epidemiology and Medical Technology Assessment, Maastricht University Medical Centre, Maastricht, The Netherlands; 15 GROW - School for Oncology and Developmental Biology, Maastricht University Medical Centre, Maastricht, The Netherlands; 16 Department of Surgical Oncology, Maastricht University Medical Centre, P.O. Box 5800 6202 AZ, Maastricht, The Netherlands

## Abstract

**Background:**

Trials failed to demonstrate additional value of completion axillary lymph node dissection in case of limited sentinel lymph node metastases in breast cancer patients undergoing breast conserving therapy. It has been suggested that the low regional recurrence rates in these trials might partially be ascribed to accidental irradiation of part of the axilla by whole breast radiation therapy, which precludes extrapolation of results to mastectomy patients. The aim of the randomized controlled BOOG 2013–07 trial is therefore to investigate whether completion axillary treatment can be safely omitted in sentinel lymph node positive breast cancer patients treated with mastectomy.

**Design:**

This study is designed as a non-inferiority randomized controlled multicentre trial. Women aged 18 years or older diagnosed with unilateral invasive clinically T1-2 N0 breast cancer who are treated with mastectomy, and who have a maximum of three axillary sentinel lymph nodes containing micro- and/or macrometastases, will be randomized for completion axillary treatment versus no completion axillary treatment. Completion axillary treatment can consist of completion axillary lymph node dissection or axillary radiation therapy. Primary endpoint is regional recurrence rate at 5 years. Based on a 5-year regional recurrence free survival rate of 98 % among controls and 96 % for study subjects, the sample size amounts 439 per arm (including 10 % lost to follow-up), to be able to reject the null hypothesis that the rate for study and control subjects is inferior by at least 5 % with a probability of 0.8. Results will be reported after 5 and 10 years of follow-up.

**Discussion:**

We hypothesize that completion axillary treatment can be safely omitted in sentinel node positive breast cancer patients undergoing mastectomy. If confirmed, this study will significantly decrease the number of breast cancer patients receiving extensive treatment of the axilla, thereby diminishing the risk of morbidity and improving quality of life, while maintaining excellent regional control and without affecting survival.

**Trial registration:**

The BOOG 2013–07 study is registered in the register of ClinicalTrials.gov since April 10, 2014, Identifier: NCT02112682.

**Electronic supplementary material:**

The online version of this article (doi:10.1186/s12885-015-1613-2) contains supplementary material, which is available to authorized users.

## Background

For a long time the standard procedure to assess the axillary lymph node status in breast cancer was an axillary lymph node dissection (ALND). This operation is associated with significant morbidity and a decrease in quality of life [1, 2]. The therapeutic benefit of this operation – improving overall survival and maintaining regional control – has been questioned in several trials.

The NSABP B-04 trial was initiated in 1971 and randomized clinically node negative breast cancer patients to radical mastectomy, mastectomy followed by axillary radiation therapy, or mastectomy followed by a delayed ALND after the development of palpable lymphadenopathy during follow-up [3]. The ALND specimen of patients in the radical mastectomy group contained lymph node metastases in 40 % of the patients. Nevertheless, this trial demonstrated that omitting primary axillary treatment of occult positive lymph nodes in patients with a clinically node negative status did not affect distant disease free- and overall survival, even after 25 years of follow-up and without the use of adjuvant systemic or radiation therapy. A delayed ALND was performed in 18.6 % of the patients in the mastectomy-only group, which is less than half of the patients with occult positive lymph nodes based on the radical mastectomy group [3]. The ‘wait-and-see’ policy therefore prevented axillary overtreatment in the majority of patients. Despite these favourable results, ALND remained to be the standard procedure to assess the axillary lymph node status, partly due to adjuvant systemic therapy that appeared to be mainly beneficial for node-positive breast cancer patients.

In the past 15 years, the sentinel lymph node biopsy (SLNB) has become the standard, less invasive technique for nodal staging of clinically node negative breast cancer patients [4]. A completion ALND was, until recently, routinely performed in patients with a metastasis in the sentinel lymph node (SLN) [5].

The AMAROS trial demonstrated that axillary and periclavicular radiation therapy could safely replace completion ALND in patients with clinically T1-2 breast cancer, no palpable lymphadenopathy and a positive SLN, without compromising the 5-year regional recurrence rate, disease free- and overall survival [6]. Patients in the AMAROS trial were treated with breast conserving therapy in 82 %, with mastectomy in 18 %, and adjuvant systemic therapy in 90 % of the cases. At five years, a significant lower lymphedema rate based on arm circumference measurements was observed, favouring the radiation therapy group.

Two recent trials further suggest that completion ALND might be safely omitted [7, 8]. The ACOSOG Z0011 trial randomized patients with clinically T1-2 breast cancer, no palpable lymphadenopathy and 1–2 macrometastatic SLNs, who were treated with breast conserving therapy, to completion ALND or watchful waiting [7]. The ALND specimen of 27 % of the patients in the completion ALND group contained additional lymph node metastases beyond the SLN, but omitting the completion ALND in the watchful waiting arm did not result in an inferior regional recurrence rate, disease free- or overall survival [7, 9]. Findings of the ACOSOG Z0011 are supported by results of the IBCSG 23–01 trial, which revealed that further axillary treatment can be safely omitted after the detection of a micrometastasis in the SLN [8]. All patients in the IBCSG 23–01 trial had a clinically T1-2 status and no palpable lymphadenopathy, 91 % was treated with breast conserving therapy, 9 % with mastectomy and 97 % with adjuvant systemic therapy.

The clinically node negative patients in the AMAROS, ACOSOG Z0011 and IBCSG 23–01 trial were selected by physical examination of the axilla. In the Netherlands, an axillary ultrasound next to physical examination is routinely performed for preoperative lymph node staging, combined with tissue sampling in case of a suspicious lymph node [5]. The ESMO breast cancer guideline also describes that an ultrasound of the regional lymph nodes should be included in the diagnostic work-up of breast cancer patients, and recommends not to perform an SLNB when axillary lymph node involvement is proven on ultrasound-guided biopsy [10]. The accuracy of physical examination of the axilla for preoperative lymph node staging is low, with a sensitivity of up to 32 % for detecting axillary metastases [11, 12]. The sensitivity of axillary ultrasound combined with tissue sampling if indicated is 50–55 % [13, 14]. Furthermore, patients with a more favourable tumour load are selected when an axillary ultrasound is performed, as the total number of nodal metastases is significantly lower after a negative axillary ultrasound than after negative physical examination [15]. In addition, a negative axillary ultrasound accurately excludes advanced nodal disease (≥4 lymph node metastases) with a negative predictive value of 93–96 % [16, 17]. The performance of an axillary ultrasound for preoperative nodal staging might therefore be beneficial when incorporating the omission of completion axillary treatment in patients with SLN metastases into daily practice [18].

The AMAROS, ACOSOG Z0011 and IBCSG 23–01 trial were underpowered, as events occurred less common than anticipated [6–8]. Low regional recurrence rates in the study arms of these trials of 1.0 %, 0.9 % and 1.1 %, respectively, might be due to treatment of most patients with breast conserving therapy and adjuvant systemic therapy. Whole breast radiation therapy in the context of breast conserving therapy is known to decrease the regional recurrence rate, most likely caused by accidental irradiation of part of the axilla [19–21]. However, biology and systemic therapy also play a role in achieving low regional recurrence rates. The NSABP B-04 trial demonstrated that less than half of the patients with occult nodal metastases develop clinically detectable lymph nodes, while none of the patients received adjuvant systemic therapy [3]. Reported pathologic complete response rates for axillary lymph node metastases following primary systemic therapy of up to 40 %, demonstrate that systemic therapy can eradicate lymph node metastases [22, 23].

The non-inferior regional recurrence-, disease free- and overall survival rates in the ACOSOG Z0011 and IBCSG 23–01 trial imply that extensive surgical treatment of lymph node metastases with a completion ALND is not of added value for breast cancer patients with a clinically T1-2 status, no palpable lymphadenopathy, limited SLN metastases, who are treated with breast conserving therapy and adjuvant systemic therapy [7, 8]. Results of the ACOSOG Z0011 and IBCSG 23–01 trial cannot be extrapolated to SLN positive patients treated with mastectomy, as these patients do not routinely receive adjuvant radiation therapy.

Therefore, we propose the randomized controlled BOOG 2013–07 trial to prove that completion axillary treatment can be safely omitted in breast cancer patients with a clinically T1-2 status, a negative axillary ultrasound and limited SLN metastases, who are treated with a mastectomy. We aim to decrease the number of breast cancer patients receiving overtreatment of the axilla, to diminish the risk of morbidity and to improve quality of life, while maintaining excellent regional control and without affecting survival.

### Main study objectives

The main aim of the BOOG 2013–07 study is to investigate whether omitting completion axillary treatment is non-inferior to completion axillary treatment in terms of the 5 and 10-year regional recurrence rate, in breast cancer patients with a clinically T1-2 status, a negative axillary ultrasound and limited SLN metastases, who are treated with a mastectomy. Secondary objectives that are assessed during a follow-up of 10 years include the assessment of quality of life, distant-disease free survival, overall survival, local recurrence rate, contralateral breast cancer, administration of adjuvant radiation therapy, and delayed axillary treatment.

## Methods

### Study design

The BOOG 2013–07 is a Dutch non-inferiority randomized controlled multicentre trial. Patients with clinically T1-2 invasive breast cancer, negative axillary ultrasound and limited SLN metastases, who are treated with mastectomy, are randomized to completion axillary treatment or no completion axillary treatment. Outcome will be evaluated after 5 and 10 years of follow-up. This study will be performed in 43 centres in the Netherlands. The study was conducted in accordance to the standards of Good Clinical Practice, in agreement with the Declaration of Helsinki and with Dutch law in general and with the Medical Research Involving Human Subjects Act (in Dutch: Wet Medisch-wetenschappelijk Onderzoek met mensen) in particular. This study was approved by the medical ethics committee of the Netherlands Cancer Institute - Antoni van Leeuwenhoek Hospital (PTC14.0032/M14CAT). The Board of Directors approved initiation of the study in current participating centres that are open for accrual (Additional file 1). The BOOG 2013–07 trial is registered at ClinicalTrials.gov (NCT02112682).

### Study population

Women aged 18 years or older diagnosed with clinically T1-2 N0 invasive breast cancer, who are treated with a mastectomy and who have a minimum of one micrometastatic and a maximum of three macrometastatic axillary SLNs, are eligible for inclusion. Clinically N0 is defined as no signs of axillary lymph node metastases at physical examination and preoperative axillary ultrasound (or negative cyto-/histopathology). Primary systemic therapy and primary and secondary breast reconstructions are allowed.

Exclusion criteria include the following: SLNs containing only isolated tumour cells (<0.2 mm); solitary parasternal SLN metastasis; bilateral breast cancer; evidence of metastatic disease; history of invasive breast cancer; previous treatment of the axilla with surgery or radiation therapy (except surgery for hidradenitis suppurativa or for other superficially located skin lesions, such as naevi); pregnancy or lactation; other prior malignancies, except successfully treated malignancies that occurred more than five years before randomization, and except successfully treated basal cell and squamous cell skin cancer, and carcinoma in situ of the breast or cervix.

#### Axillary ultrasound

Axillary ultrasound is standard of care in the Netherlands for preoperative nodal staging of breast cancer patients [5]. The following criteria are used during ultrasound of axillary level 1–3 to identify positive lymph nodes: long to short axis ratio of <2 (i.e. round), diffuse or focal cortical thickening, effacement or replacement of the fatty hilum, and/or nonhilar blood flow (using Doppler ultrasound, if detectable). As described in the Dutch breast cancer guideline, cortical thickening of more than 2.3 mm is considered as the optimal cut-off point to perform fine-needle aspiration biopsy [5]. Additionally, a subjective assessment of thickening can be made by the radiologist during real-time imaging, similar to the studies by Koelliker et al., Abe et al., and Neal et al. [16, 24, 25]. Fine-needle aspiration biopsy or core biopsy is recommended when suspicious lymph nodes are identified. In case of two or more abnormal lymph nodes, the lymph node with the most suspicious findings is selected for tissue sampling.

#### Sentinel lymph node biopsy

For the SLNB, Technetium-99 m Nanocolloid will be injected into breast parenchymal tissue surrounding the tumour, biopsy cavity or periareolar, followed by lymphoscintigraphic images. The SLN (s) will be identified during surgery by using the following triple technique: lymphoscintigraphic images, blue dye and a gamma probe. Palpation of the axilla after removal of the SLN (s) is performed to identify and remove suspicious (non-) SLN (s).

As a minimal requirement for pathological assessment, each SLN is examined at three histological levels (500-μm intervals). On each level two parallel sections are performed, one for haematoxylin and eosin (H&E) staining and one for immunohistochemical (IHC) staining. IHC staining is done only when H&E staining is negative, and is performed for markers containing at least cytokeratin 8 and 18 (e.g. CAM 5.2, NCL5D3). Lymph nodes submitted for pathological examination, which are marked by the surgeon as non-SLNs are examined with H&E and if negative with cytokeratin IHC staining. The exact diameter of each metastasis must be determined, as well as describing the occurrence of extranodal growth. Isolated tumour cells (<0.2 mm) are considered as SLN negative.

#### Mastectomy

A mastectomy is defined as the surgical removal of all glandular breast tissue. The size of the primary tumour is determined during pathological assessment. The hormone receptor status is determined by IHC staining and is considered positive if ≥10 % of the cells stain positive. HER2neu status is determined by IHC and in case of 2+ determined by CISH or FISH. Histological tumour grading is assessed according to the modified Bloom-Richardson grading system. The presence of multifocality is defined as foci or carcinoma separate from the primary tumour. The histological tumour type is defined according to the World Health Organization. Presence of lymphovascular invasion is defined as one or more tumour cells in a lymphatic or vascular structure.

The modified Bloom-Richardson grading system consists of three components of the tumour morphology and a score of 1, 2 of 3, is assigned to each of these components: the extent of tubule formation (1 = >75 %; 2 = 10–75 %; 3 = <10 %), the nuclear polymorphism (1 = comparable to normal epithelium; 2 = enlarged, vesicular, small nucleoli; 3 = polymorphic, vesicular, large nucleoli) and mitotic activity defined as the number of mitoses per 2 mm^2^ (1 = 0–7 mitoses per 2 mm^2^; 2 = 8–12 mitoses per 2 mm^2^; 3 = ≥13 mitoses per 2 mm^2^). The histological grade is determined by the sum of these scores, with grade I for the scores 3–5, II for 6–7, and III for 8–9.

### Consent and randomization

Eligible patients will be informed about the study aims, study procedures, possible adverse events, mechanism of treatment allocation, and their rights and responsibilities. After written informed consent is obtained, patients will be randomized between completion axillary treatment (control arm) and no completion axillary treatment (study arm).

Stratification factors for randomization include the following: age (≤50, 50 ≤ 75, >75), oestrogen receptor status (positive vs. negative), HER2neu status (amplified vs. not-amplified), lymph node metastasis (micro- vs. macrometastasis), clinical tumour size prior to any treatment (<3 cm vs. ≥3 cm), grading (grade I-II vs. III - according to modified Bloom-Richardson grading system), primary systemic therapy and participating centre.

### Completion axillary treatment

Completion axillary treatment in the control arm can consist of a completion ALND or axillary radiation therapy in accordance to the Dutch breast cancer guideline [5]. Axillary radiation therapy can either be of axillary level 1 and 2 (i.e. the regions that would be operated upon if an ALND would be performed), or radiation therapy of axilla level 1–3 and periclavicular nodes (i.e. conform the regions that were irradiated in the AMAROS trial). Each participating centre states on beforehand which radiation strategy they follow for which patient categories.

### Radiation therapy

#### Chest wall irradiation

Radiation therapy of the chest wall after mastectomy is indicated in specific circumstances depending on the Dutch and local protocols and therefore not an exclusion criterion in this study. According to the Dutch breast cancer guideline, postoperative radiation therapy after mastectomy can be considered in patients with 1–3 axillary lymph nodes containing metastatic disease with at least one risk factor. Risk factors include angioinvasive growth, grade III tumours, tumour size of ≥3 cm and/or age ≤ 40 years [5]. The indications for radiation therapy will be clearly defined for each participating centre to prevent a low-threshold for chest wall irradiation in study arm B.

#### Dose and fractionation for chest wall and axilla

A fractionation scheme equivalent to 25 × 2 Gy, 5 fractions per week is applied; i.e. schemes of 15–16 × 2.66 Gy, 5 fractions per week are allowed as well. In case of an irradical resection a boost is given to the tumour bed, equivalent to 7–13 × 2 Gy. A simultaneous integrated boost is recommended, with high fraction size not exceeding 2.67 Gy.

#### Delineation of chest wall and axilla

Delineation of target volumes is performed using the ESTRO guidelines of Offerson et al. [26]. Delineation of all target volumes, including the thoracic wall and heart and lungs is obligatory. Delineation of other normal structures is optional. To allow adequate evaluation of the radiation in the axillary nodal regions, delineation of axillary level 1, 2, Rotter nodes, 3 and 4 is obligatory, also in case of chest wall irradiation alone.

#### Radiation technique and dose distribution

The dose in the Planning Target Volume of the chest wall with or without axillary and periclavicular nodes must be between 95–107 % of the prescribed dose. If the distance between the skin and the pectoral muscle is < 5 mm, it is allowed to use tissue-equivalent material to increase the superficial dose. The mean lung dose should be < 5 Gy in case of tangential fields only, and < 7.5 Gy in case of locoregional irradiation. The heart volume receiving > 25 Gy should be < 20 %; the Mean Heart Dose should preferably be below 3 Gy, and should certainly not exceed 5 Gy. If lung or heart constraints cannot be met, some underdose in the thoracic wall target volume can be accepted to reach the constraints, provided that the quadrant where the primary tumour was localized is adequately covered. Respiratory control techniques to reduce heart dose are highly recommended for left sided breast cancer patients. The dose in the brachial plexus should be kept below an equivalent of 60 Gy in 30 fractions. The minimum, maximum and mean dose of the axilla level 1, 2, Rotter nodes, 3 and 4 must always be recorded for evaluation purposes, even in case only chest wall radiation therapy is applied.

### Systemic therapy

The indication for systemic therapy is determined for the individual patient according to the Dutch breast cancer guideline and multidisciplinary approach. Primary systemic therapy in clinically T1-2 (pre-systemic therapy) patients is no exclusion criterion.

### Follow-up

During the 10-year follow-up period, outpatient clinic visits take place annually with physical examination of the axilla. A mammography is performed annually in the first five years of follow-up. In year six to ten, a mammography is performed annually in patients aged ≤60 years or once every two years in patients aged >60 years. Additional diagnostic imaging is performed on indication. An axillary ultrasound is performed in patients with a clinical suspicion of axillary lymph node metastases during follow-up. If an axillary lymph node metastasis is confirmed by tissue sampling, staging for distant metastatic disease is performed in accordance to the Dutch breast cancer guideline. In patients with a clinical suspicion of distant metastatic disease during follow-up, staging for metastatic disease is performed, in combination with physical examination of the axilla for the detection of possible axillary lymph node metastases, followed only by an axillary ultrasound in patients with a clinical suspicion of axillary lymph node metastases.

### Quality of life

Quality of life will be assessed with a set of questionnaires. The first set is provided pre-randomisation for baseline measurement, and the following are provided sequentially post-randomization at 6 months, and at 1, 2, 5 and 10 years. Patients are eligible for evaluation only when at least the pre-randomisation questionnaire and the subsequent questionnaire is completed. The set of questionnaires consist of the EORTC QLQ-C30 and QLQ-BR 23 questionnaire, the Lymph-ICF, STAI-trait and NEO-FFI questionnaire [27–30]. The combination of these questionnaires will provide information on the general and breast cancer specific quality of life, subjective morbidity, and anxiety and personality traits that might influence the outcome of quality of life [31].

### Adverse events

Adverse events (AEs) are defined as any undesirable experience occurring to a subject during the study, whether or not considered related to the protocol treatment. All AEs reported spontaneously by the subject or observed by the investigator or his staff will be recorded. Predefined AEs concerning axillary morbidity include seroma, postoperative haemorrhage, wound complication/infection, lymphedema of the arm, lymphedema of the chest wall, neuralgia, paraesthesia, decreased range of motion of the arm or shoulder, muscle weakness of the arm or shoulder, and pain in the arm or shoulder. The severity of the AE is graded according to the NCI/CTCAE 4.0 grading criteria into mild, moderate, or severe, in combination with the degree of limitation in activities of daily living.

A serious adverse event (SAE) is defined as an untoward medical occurrence or effect related to mastectomy, SLNB, completion ALND or axillary radiation therapy that results in death, hospitalisation or prolongation of existing inpatients hospitalisation, or surgery. Other adjuvant treatment is not considered protocol treatment. The local investigator of the participating centre where the SAE occurs is responsible to report the SAE to the central data centre within 24 h. The principal investigators of the study are responsible for SAE assessment and reporting to the accredited medical ethics committee within 15 days. For fatal or life threatening cases, the term will be maximal 7 days for a preliminary report with another 8 days for completion of the report. All SAEs will be followed until they have abated, or until a stable situation has been reached. Depending on the event, follow-up may require additional tests or medical procedures as indicated and/or referral to the general physician or a medical specialist.

### Statistics

#### Endpoints

Regional recurrence rate is the primary endpoint in this study. Secondary endpoints include number of delayed axillary treatment, distant-disease free survival, overall survival, local recurrence rate, other-regional recurrence rate, contralateral breast cancer rate, percentage difference in the administration of postoperative radiation therapy, axillary morbidity rate and quality of life. The events included in the definitions of the different recurrences are provided in Table 1, and are based on the Maastricht Delphi Consensus on Event Definition by Moossdorff et al. [32, 33]. Pathological confirmation of a regional recurrence is mandatory, and recommended in case of other suspicious lesions. All cases with a lesion that is highly suspicious for tumour recurrence on imaging, but not accessible for tissue sampling are presented to the Data Safety Monitoring Board (DSMB) for an independent review.

**Table 1:** Definition of a regional, other-regional, local and distant recurrence

Classification	Events included
Regional recurrence	Recurrence in an ipsilateral axillary-, infraclavicular-, or supraclavicular lymph node
Other-regional recurrence	Recurrence in an ipsilateral internal mammary lymph node
Local recurrence	Recurrence in skin or subcutaneous tissue on the ipsilateral chest wall
Distant recurrence	Recurrence in any other location, including recurrence involving the
	sternal bone, or contralateral lymph nodes

Time to event endpoints are defined as the time interval between the date of randomization and the date of first suspicion of the predefined recurrence, or the date of death, whichever comes first, measured in days. Patients in whom recurrence is not observed and are still alive are censored at the date of last follow-up. Death from breast cancer and its treatment, death from a second primary invasive non-breast cancer, and death from other- or an unknown cause are recorded.

Administration of postoperative radiation therapy is registered and the percentage difference between both study arms is recorded. Axillary morbidity rate will be assessed using a validated questionnaire and by predefined adverse events that are recorded by the treating physician. Quality of life will be assessed using validated questionnaires.

#### Sample size

Prior data indicate a 5-year regional recurrence free survival rate of 98 % for the control patient group, and a regional recurrence free survival rate of 96 % is expected for the experimental patient group. A difference of no more than 5 % (delta = 5 %) is considered acceptable, when taking in account the higher morbidity rate caused by completion axillary treatment in the control arm. The expected regional recurrence free survival rates and delta result in a sample size of 399 per arm. Therefore, we will need to study 399 experimental subjects and 399 control subjects to be able to reject the null hypothesis that the rate for experimental and control subjects is inferior by at least 5 % with a probability of 0.8. When taking in account a lost to follow-up rate of 10 %, 878 patients need to be randomized. An annual accrual of 324 patients can be achieved, based on the incidence of women diagnosed with invasive breast cancer in the Netherlands, the rate of patients that is operated on primarily (excluding patients treated with chemo- or hormonal therapy only, or with metastatic disease and frail elderly), the rate of patients with a positive SLN, treatment with mastectomy, the 43 participating hospitals, and an expected accrual rate of 30 %. Therefore, three years will suffice to include the 878 patients.

#### Interim analysis

For the interim analysis, the sample size was recalculated with doubling of the delta from 5 to 10 % to reduce the risk that the study is incorrectly aborted prematurely. Together with a lost to follow-up rate of 10 %, the total number of patients included for this analysis amounts 125.

An independent statistician will perform the interim analysis after a two-year follow-up of the first 125 included patients, because the total accrual is expected to complete within 3 years and because most regional recurrences occur within two years after initial treatment. According to the Haybittle-Peto boundary, a *P* value of 0.001 or less is considered statistically significant for this analysis [34]. Results of the interim analysis are reported to the DSMB.

#### Data safety monitoring board

The independent DSMB comprises a surgeon, medical oncologist, radiation oncologist and a statistician. The DSMB will meet annually to discuss the occurrence and nature of adverse events occurring during the study, initially at a 1-year interval. During the study, the DSMB may decide to change the frequency of discussion. All cases with a lesion that is highly suspicious for tumour recurrence on imaging, but not accessible for histology or cytology are presented to the DSMB for an independent review. Further, the DSMB is informed about the results of the interim analysis for further interpretation. DSMB recommendations are sent to the principal investigators. Should the principle investigators decide not to fully implement the DSMB recommendations, the principle investigators will send the recommendation to the accredited medical ethics committee, including a note to substantiate why (part of) this recommendation will not be followed.

#### Stopping rule

The right to discontinue the study prior to inclusion of the intended number of subjects is reserved to the principle investigators, but intends only to exercise this right for valid scientific or administrative reasons such as a negative advice for continuing the study by the DSMB, or disappointing accrual so that the total enrolment of 878 patients seems not feasible within the planned study period.

#### Final analysis

Primary and secondary endpoints will be analysed per protocol and in the intention to treat population after 5 and 10 years of follow-up. Primarily, uncorrected chi-squared statistics will be used to evaluate the null hypothesis. The chi-square test will be based on the Kaplan-Meier estimator, in case of censored data. Additionally, cox proportional hazards models and Kaplan Meier estimates will be used to analyse the outcome of both groups and to assess the univariable and multivariable association between prognostic variables, treatment and events, using the stratification factors. All statistical tests are 1-sided and a *P* value of 0.05 or less is considered statistically significant.

## Additional file

Additional file 1: **Participating centres.** (DOCX 20 kb)

## Abbreviations

AE: Adverse event
ALND: Axillary lymph node dissection
DSMB: Data Safety Monitoring Board
H&E: Haematoxylin and eosin
IHC: Immunohistochemistry
SAE: Serious adverse event
SLN: Sentinel lymph node
SLNB: Sentinel lymph node biopsy
