# Supplementary material for: The value of completion axillary treatment in sentinel node positive breast cancer patients undergoing a mastectomy: a Dutch randomized controlled multicentre trial (BOOG 2013-07)
Source: BMC Cancer. 2015 Sep 3;15:610. doi: 10.1186/s12885-015-1613-2 (PMC4559064; doi:10.1186/s12885-015-1613-2)
Supplement: Additional file 1: — Participating centres. (DOCX 20 kb) [file 12885_2015_1613_MOESM1_ESM.docx]

|  | **Participating centres** |
| --- | --- |
| 1 | Albert Schweitzer Hospital |
| 2 | Amphia Hospital |
| 3 | Antonius Hospital |
| 4 | Atrium Medical Centre |
| 5 | Bronovo Hospital |
| 6 | Canisius-Wilhelmina Hospital |
| 7 | Catharina Hospital Eindhoven |
| 8 | De Sionsberg |
| 9 | Deventer Hospital |
| 10 | Diakonessenhuis Utrecht |
| 11 | Gelre Hospital |
| 12 | Groene Hart Hospital |
| 13 | Haga Hospital |
| 14 | Ikazia Hospital |
| 15 | Isala Clinic |
| 16 | Kennemer Gasthuis |
| 17 | LangeLand Hospital |
| 18 | Laurentius Hospital |
| 19 | Maastricht University Medical Centre |
| 20 | Martini Hospital |
| 21 | Máxima Medical Centre |
| 22 | Meander Medical Centre |
| 23 | Medical Centre Alkmaar |
| 24 | Medical Centre Haaglanden |
| 25 | Medical Spectrum Twente |
| 26 | Netherlands Cancer Institute |
| 27 | Onze Lieve Vrouwe Gasthuis |
| 28 | Orbis Medical Centre |
| 29 | Radboud University Medical Centre |
| 30 | Reinier de Graaf Groep |
| 31 | Rijnstate Hospital |
| 32 | Rivierenland Hospital |
| 33 | Sint Antonius Hospital |
| 34 | Sint Elisabeth Hospital |
| 35 | Streekziekenhuis Koningin Beatrix |
| 36 | Tergooi Hospital |
| 37 | University Medical Centre Groningen |
| 38 | University Medical Centre Utrecht |
| 39 | VieCuri Medical Centre |
| 40 | Waterland Hospital |
| 41 | Westfries Gasthuis |
| 42 | Hospital Gelderse Vallei |
| 43 | Ziekenhuisgroep Twente |
